# *Class I KNOX* Is Related to Determinacy during the Leaf Development of the Fern *Mickelia scandens* (Dryopteridaceae)

**DOI:** 10.3390/ijms21124295

**Published:** 2020-06-16

**Authors:** Rafael Cruz, Gladys F. A. Melo-de-Pinna, Alejandra Vasco, Jefferson Prado, Barbara A. Ambrose

**Affiliations:** 1 Instituto de Botânica, Av. Miguel Estéfano 3687, São Paulo (SP) CEP 04301-902, Brazil; jprado.01@uol.com.br; 2 Instituto de Biociências, Universidade de São Paulo, Rua do Matão 277, São Paulo (SP) CEP 05422-971, Brazil; gfmpinna@usp.br; 3 Botanical Research Institute of Texas, 1700 University Drive, Fort Worth, TX 76107-3400, USA; avascog@gmail.com; 4 UNESP, IBILCE, Depto. de Zoologia e Botânica, Rua Cristóvão Colombo, 2265, São José do Rio Preto (SP) CEP 15054-000, Brazil; 5 The New York Botanical Garden, 2900 Southern Blvd, Bronx, NY 10458-5126, USA; bambrose@nybg.org

**Keywords:** apical meristems, *Class I KNOX* genes, compound leaves, determinacy, Dryopteridaceae, ferns, leaf development, pinna development, shoot development

## Abstract

Unlike seed plants, ferns leaves are considered to be structures with delayed determinacy, with a leaf apical meristem similar to the shoot apical meristems. To better understand the meristematic organization during leaf development and determinacy control, we analyzed the cell divisions and expression of *Class I KNOX* genes in *Mickelia scandens*, a fern that produces larger leaves with more pinnae in its climbing form than in its terrestrial form. We performed anatomical, in situ hybridization, and qRT-PCR experiments with *histone H4* (cell division marker) and *Class I KNOX* genes. We found that *Class I KNOX* genes are expressed in shoot apical meristems, leaf apical meristems, and pinnae primordia. During early development, cell divisions occur in the most distal regions of the analyzed structures, including pinnae, and are not restricted to apical cells. Fern leaves and pinnae bear apical meristems that may partially act as indeterminate shoots, supporting the hypothesis of homology between shoots and leaves. Class *I KNOX* expression is correlated with indeterminacy in the apex and leaf of ferns, suggesting a conserved function for these genes in euphyllophytes with compound leaves.

## 1. Introduction

Vascular plant organs are classically defined based on their position; on their tissue organization (symmetry axes and vascular tissue); and on the presence, position, and activity of their meristems [1,2]. With these criteria, leaves are lateral determinate organs generally with an abaxial-adaxial asymmetry, and these features seem to generally apply well to leaves in seed plants. On the other hand, shoots are characterized by indeterminacy and are marked by the expression of *Class I KNOTTED-LIKE HOMEOBOX* (*KNOX*) genes in the shoot apical meristem (SAM) [3]. This class of genes belongs to the superclass three amino acid loop extension (*TALE*) of homeodomain proteins [4,5]. The downregulation of *Class I KNOX* is one of the first indications of the development of a determinate leaf primordium in seed plants [6,7]. Plants with defective *Class I KNOX* genes may be unable to maintain the SAM, as indicated by the mutants *shoot meristemless* (STM) in *Arabidopsis thaliana* (L.) Heynh. [8,9] and by mutants carrying malfunctioning alleles such as *knotted1* in maize that have defective branching and lateral organ formation [6,10]. In most vascular plants, when *Class I KNOX* genes are naturally expressed in the leaf primordium, the resulting morphology usually will be a compound leaf, as demonstrated by Bharathan et al. [7] in an extensive exploration of different groups (including many angiosperms, the cycad *Zamia floridana* A. DC., and the fern *Anogramma chaerophylla* (Desv.) link). This rule seems to have few known exceptions, except for legumes [11]. By analyzing *Cardamine hirsuta* L., a crucifer related to *Arabidopsis* with dissected leaves, Hay and Tsiantis [12] concluded that the expression of the STM homolog in the leaf primordium delays differentiation pathways, allowing leaflet initiation, while *Arabidopsis thaliana* produces simple leaves due to the exclusion of *Class I KNOX* expression from the leaf primordium. *Class I KNOX* genes have been found to be expressed and have a function in the meristematic regions of various organs in seed plants, and as such have been related to indeterminacy [10,13,14]. Thus, compound leaves can be interpreted as structures with a delayed determinacy during their development, and Class *I KNOX* facilitates leaflet formation [7,15,16].

Fern leaves are different from most seed plant leaves. For example, unlike seed plants, many fern leaves have a leaf apical meristem (LAM). In ferns, the LAM is responsible for a transient indeterminacy during leaf development, usually producing lateral pinnae during a longer period than the regular compound leaf of a seed plant. The LAM and SAM structure of ferns is also remarkably unique, in that they both have a distinctive prominent apical cell and a peripheral zone that together compose the entire LAM or SAM [17,18,19,20,21,22]. Some ferns in the orders Marattiales and Ophioglossales do not have only a single apical cell but instead have a group of apical initial cells [18,23]. However, the marattioid fern *Angiopteris lygodiifolia* Rosenst. has only a single initial apical cell in its shoot apex, as detected in a more detailed analysis [24]. Another key difference between fern and seed plants’ leaf developments is that fern leaves mainly develop from the LAM and a marginal meristem (MM) composed of marginal and submarginal initials [21,22].

The expression analyses of two *Class I KNOX* genes in *Elaphoglossum peltatum* (Sw.) Urb. f. *peltatum* (Dryopteridaceae) characterize it as having a multicellular SAM with an apical initial and actively dividing surrounding cells [20], supporting previous work that proposed zonation for a multicellular meristem based on anatomical evidence [17,18]. *Class I KNOX* transcripts were also detected in leaf primordia and in the multicellular apex of the ferns *Anogramma chaerophyla* [7] and *Ceratopteris richardii* Brongn. [25]. Proteins coded by this class of genes were detected in the same regions in *Osmunda regalis* L. [26]. Few details are available about the expression in the pinnae primordia or in the LAM, but the expression reported of *Class I KNOX* in the leaf primordium may be the cause of the delayed determinacy of fern leaves [26].

Meristems seem to be the key character to understand the evolution and development of fern leaves (fronds). Fern leaves resemble the indeterminate shoot by having an apical meristem, producing lateral organs and having a transient or even persistent indeterminacy (as in the genera *Lygodium* Sw., *Nephrolepis* Schott, *Salpichlaena* J. Sm. and *Jamesonia* Hook. and Grev., as reviewed in Vasco et al., [27]). These features of the fern leaf do not fit the classical morphological concept of leaves as they do for seed plants. According to these classical concepts, leaves are a distinct set of features (e.g., determinacy and flattened morphology) that perfectly exclude stem features (e.g., indeterminacy and cylindrical morphology) [28,29,30]. There is evidence that *Class I KNOX* genes are directly associated with indeterminacy and are required to make compound leaves in many cases, representing a partial homology with the shoot [15]. *Class I KNOX* genes are also an important marker of meristematic activity in fern shoots [3].

Studies of Class *I KNOX* outside of spermatophytes are still necessary to better understand their role in the development of leaves, particularly in ferns. For instance, transgenic *Arabidopsis* plants overexpressing *Ceratopteris richardii Class I KNOX* genes have lobed leaves [25]. *Ceratopteris richardii Class I KNOX* genes in *Arabidopsis* mutants only partially restore their functions, even with high levels of transgene transcripts detected in complementation assays [3]. Because Class *I KNOX* proteins act together with the other class of *TALE* proteins *BELL* to target the nucleus, it is possible that *Ceratopteris* orthologs cannot interact with different *BELL* proteins in *Arabidopsis* [3]. In angiosperms, *ARP* genes, related to the development of lateral organs, are well-known to maintain the KNOX-off state in leaves [16], but these two classes of genes seem to co-occur in meristems and leaf primordia in the fern *Osmunda regalis* [26], suggesting that other regulatory mechanisms may be present. These data reinforce the importance of new experiments about *Class I KNOX* genes in ferns to better understand their potential function and role in shaping the fern body plan.

In order to gather more information concerning leaf and apical meristem organization, we studied the expression of *Class I KNOX* genes in *Mickelia scandens* (Raddi) R. C. Moran, Labiak and Sundue (Dryopteridaceae), a leptosporangiate fern endemic to the Brazilian Atlantic Rainforest. *Mickelia scandens* has pinnate leaves that have distinct forms during its life cycle as a hemiepiphyte. It bears small leaves and thin rhizomes in its terrestrial form and longer leaves with more pinnae in the thicker rhizomes of its climbing form. This difference between terrestrial and climbing leaves is an important feature of the genus [31,32,33]. A similar morphology was described for *Mickelia guianensis* (Aubl.) R.C. Moran, Labiak and Sundue based on specimens from the French Guiana [31,32]. *Mickelia guianensis* terrestrial leaves are three times narrower and with less pinnae than climbing leaves [31,32]. These differences are well documented for several *Mickelia* species [33]. This abrupt and substantial change in form is a feature that characterizes this plant as a species with a heteroblastic development, in contraposition to homoblastic species that show only small and gradual changes during their development [34]. *Mickelia* R. C. Moran, Labiak and Sundue is also the sister group of the genus *Elaphoglossum* Schott, a genus with mostly simple-leaved species, whose plants have been the targets of evolutionary and developmental studies [20,35,36], making *Mickelia scandens* a promising model to understand leaf development in ferns with compound leaves successfully applying molecular techniques. Assuming that the differences in the size and number of pinnae between the different forms of this plant represent a differential degree of determinacy, we aim to better understand if the association of *Class I KNOX* expression with determinacy is true for ferns as it is for seed plants, since these groups are separated by c. 327 Myr of evolution [37]. Our hypothesis is that the *Class I KNOX* expression is stronger and longer in developing leaves of the climbing form and that is possibly the form that presents more delayed determinacy when compared with the terrestrial form. We also aim to better understand the meristematic structure of the developing leaves by studying cell division patterns.

## 2. Results

**Figure 1:**
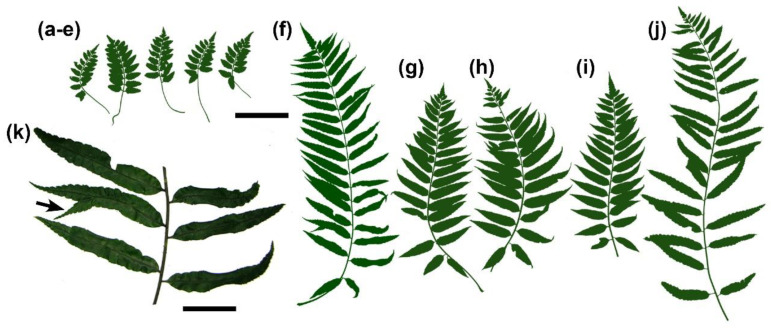
Leaves in *Mickelia scandens*. (**a**–**e**) Samples of the terrestrial form and (**f**–**j**) from the climbing form. The silhouettes are scans from actual leaves at the same scale. (**k**) Detail of the leaf of Figure 1f, with a pinna bearing a basiscopic anomalous pinnule (arrow). Bars: (**a**–**j**) 10 cm; (**k**) 4 cm.

During field collections, the clear dimorphism between the terrestrial and climbing forms of leaves was obvious (Figure 1). Although we also noticed a slight variation in size inside each of these categories, the terrestrial forms always have smaller leaves and shorter pinnae (Figure 1a–e) when compared with the climbing forms (Figure 1f–j). We also made a single observation in the field where one leaf from the climbing form had one anomalous pinna containing a basiscopic pinnule (Figure 1k).

We isolated three different *KNOX* gene homologs from *Mickelia scandens* (Supplementary Figure S1, Supplementary Table S1) using degenerate PCR. Phylogenetic analyses indicate that two of them, *MsC1KNOX1* and *MsC1KNOX2*, are closely related to known fern *Class I KNOX* genes (Supplementary Figure S2), while *MsC2KNOX1* is a *Class II KNOX* gene. We also cloned one gene that codes for *Histone H4*, *MsH4*, that was used as a positive control and cell division marker.

**Figure 2:**
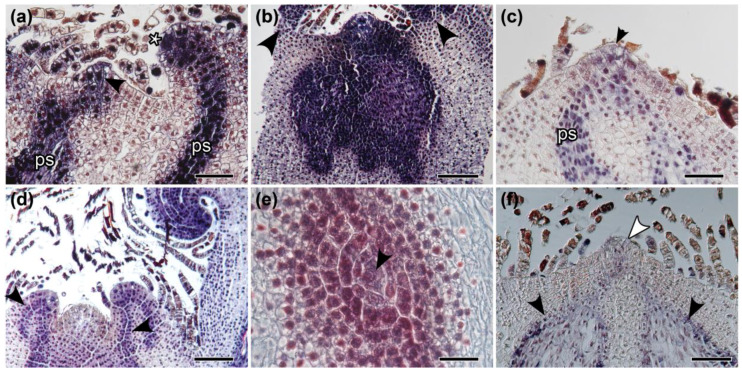
In situ hybridization of *Class I KNOX* genes and *MsH4* in shoot apices of terrestrial (Tf) and climbing (Cf) forms of *Mickelia scandens*. (**a**) Longitudinal section of Tf. Expression of *MsC1KNOX1* in the shoot apical cell (arrowhead), leaf apical cell (*), derivative cells, and procambial strands (ps). (**b**) Cf, longitudinal section. *MsC1KNOX1* expression in a peripheral zone and procambium. Leaf primordia position pointed out by arrowheads. (**c**) Longitudinal section of Tf. *MsC1KNOX2* expression in the shoot apical cell (arrowhead), derivative cells, and procambial strands (ps). (**d**) Longitudinal section of Cf. In this apex, *MsC1KNOX2* expression is not detected in the shoot apical cell. There are procambial strands (arrowheads) connecting the SAM and the leaf primordia. (**e**) Transverse section of Tf. *MsC1KNOX2* expression in a large apical cell (arrowhead) and in the surrounding derivative cells. (**f**) Longitudinal section of Cf. Scattered *MsH4* expression in the SAM (white arrowhead) and in the developing vascular system (black arrowheads). Bars: (**a**) 100, (**b**) 200, (**c**) 75, (**d**) 200, (**e**) 50, (**f**) 125 µm.

**Figure 3:**
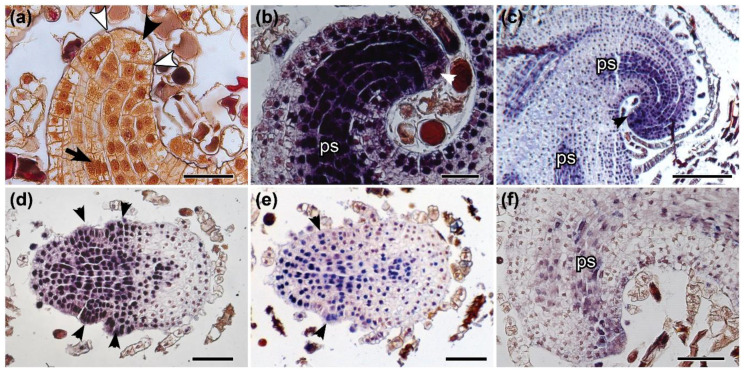
Investigations of leaf development in terrestrial (Tf) and climbing (Cf) forms in *Mickelia scandens* by anatomical and expression analyses. Longitudinal sagittal sections in (**a**–**c**,**f**); paradermal sections in (**d**,**e**). (**a**) Anatomical section of leaf apex bearing apical cell with a distal lenticular face (black arrowhead) and two dividing proximal cutting faces (their limits pointed out by white arrowheads). Inner derivative cells form the procambium (arrow). In situ hybridization (**b**–**f**). (**b**) *MsC1KNOX1* (Tf) and (**c**) *MsC1KNOX2* (Cf) are expressed in the leaf apical cell (arrowhead), derivatives cells, and in procambial strands (ps). (**d**) *MsC1KNOX1* (Tf) and (**e**) *MsC1KNOX2* (Tf) are expressed in young pinnae primordia (arrowheads). (**f**) *MsH4* expression indicates cell division in multiple cells in the apical region and procambial strands (ps). Tf. Bars: (**a**,**b**) 50, (c) 200, (**d**–**f**) 100 µm.

**Figure 4:**
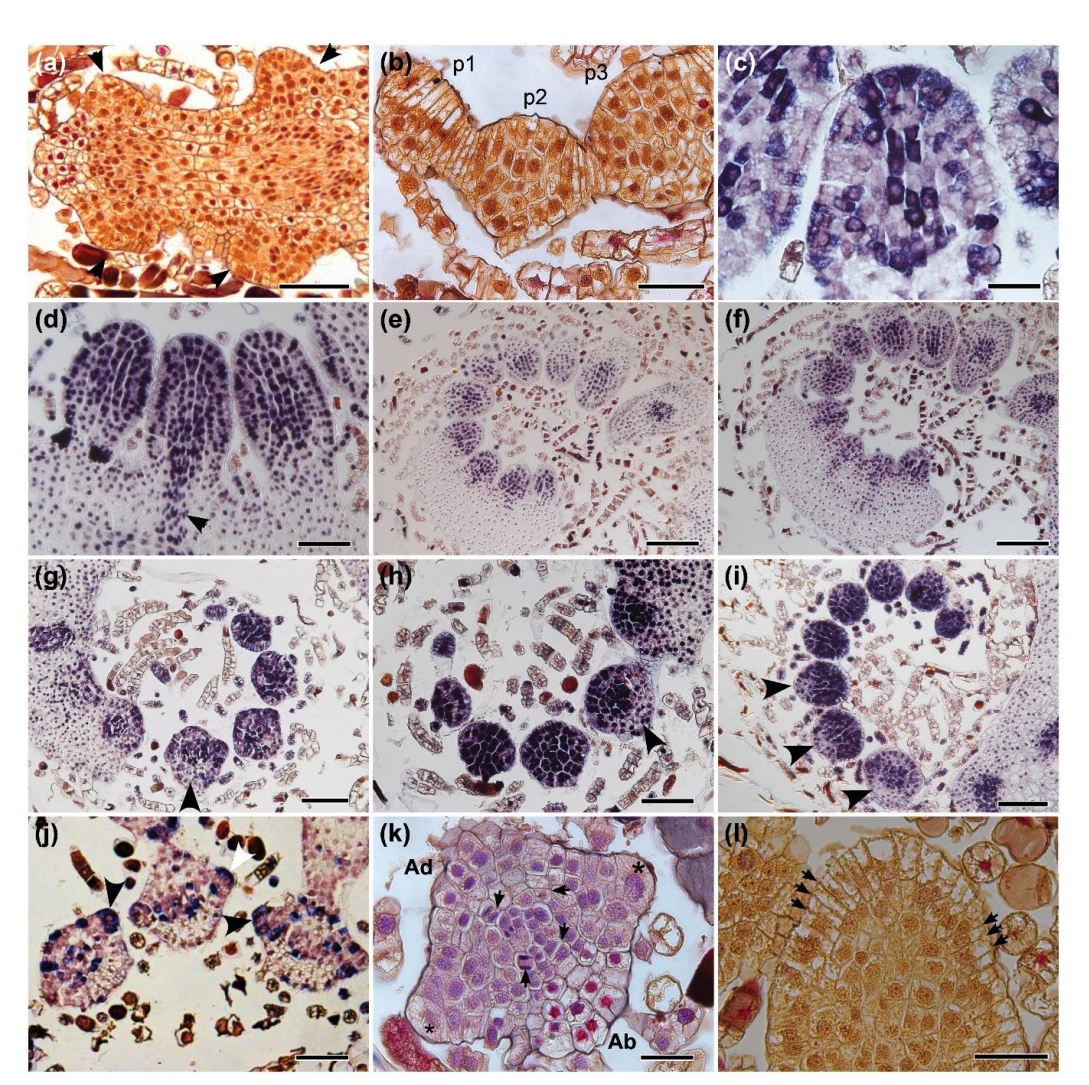
Late leaf development in terrestrial (Tf) and climbing (Cf) forms of *Mickelia scandens* by anatomical and expression analyses. Anatomical sections (**a**,**b**,**k**,**l**). (**a**) Pinnae primordia (arrowheads) emerge from the margins of the Tf leaf. (**b**) Transverse section of the youngest pinna (p1) shows grouped cells on its apex with evident periclinal divisions in Tf. The base has divisions in multiple planes, visible in older primordia (p2 and p3). In situ hybridization (**c**–**j**). (**c**) *MsH4* expression indicates cells division in multiple adjacent cells at the apex of the pinna primordium and in the central axis, where the vasculature will develop in the Tf. (**d**) As the pinnae primordium increases in size, developing vascular traces express *Class I KNOX* genes, exemplified by *MsC1KNOX1* in Cf. (**e**) *MsC1KNOX1* in Cf and (**f**) *MsC1KNOX2* in Cf are expressed in the entire young pinnae primordia. (**g**) *MsH4* in Tf, (**h**) *MsC1KNOX1* in Tf and (**i**) *MsC1KNOX2* in Cf are all expressed throughout the entire pinnae primordia, expression is gradually reduced in the abaxial side of older pinnae (arrowheads). (**j**) Cell divisions detected by *MsH4* expression in marginal cells, some indicated by arrows in Tf. (**k**) Anatomical transverse section of the pinna primordium showing marginal cells (*) with outer lenticular faces of the wall and submarginal initials, between adaxial (Ad) and abaxial (Ab) sides, and radial divisions in the center (arrows) in Cf. (**l**) Anatomical paradermal section of the pinna primordium showing rows of marginal cells, with anticlinal cutting faces, some indicated with arrows in Cf. Bars: (**a**,**d**,**h**,**j**) 100, (**b**,**c**,**k**,**l**) 50, (**e**,**f**) 200, (**g**,**i**) 150 µm.

To determine if there are differences in the patterns of expression of *Class I KNOX* genes in *Mickelia scandens* terrestrial and climbing leaf types, we assessed their expression by *in situ* hybridization (ISH). The ISH experiments show that *MsC1KNOX1* and *MsC1KNOX2* have similar temporal and spatial expression patterns during shoot, leaf, and pinnae development (Figure 2, Figure 3 and Figure 4). The *MsC1KNOX1* expression appeared stronger than *MsC1KNOX2* (Figure 2, Figure 3 and Figure 4), although ISH experiments are not quantitative. In the shoot apical meristem, *MsC1KNOX1* and *MsC1KNOX2* are expressed in the apical cell and the derivative cells in the peripheral zone in most of the experiments (Figure 2a–e). The expression of *MsC1KNOX1* and *MsC1KNOX2* is also detected in the procambial cells, which are continuous with the SAM (Figure 2a,c). *MsC1KNOX1* and *MsC1KNOX2* expression were not detected in the boundaries between the leaf primordium and the shoot apex (Figure 2b). We also detected *Class I KNOX* expression in the LAM and MM of developing leaves (Figure 2d). Occasionally, our experiments show a clear expression of *Class I KNOX* genes in the SAM peripheral zone, but no expression in the apical cell and in some of the surrounding prismatic cells (Figure 2d). This cell is bigger than derivative cells that gradually reduce in size in the SAM (Figure 2e). To compare the patterns of cell division in the developing shoot apex, we assessed the expression of *Histone H4* (*MsH4*), which has a slightly similar pattern when compared with *Class I KNOX*, being expressed in the shoot apex and in the developing vascular system (Figure 2f).

We found that during leaf development, both leaf types have similar *Class I KNOX* expression patterns in the LAM (apical initial and its derivatives, the peripheral cells). In general, the leaf primordium has a distinct apical cell with a lenticular distal face of the wall and two cutting faces (Figure 3a). The derivative cells undergo divisions, and inner cells are responsible for the establishment of the procambium (Figure 3a). In the leaf apex, we detected the expression of *MsC1KNOX1* and *MsC1KNOX2* in the LAM during leaf development (Figure 3b,c). *MsC1KNOX1* and *MsC1KNOX2* expression was also detected in the apical cell of the SAM and in the procambium (Figure 3b,c). *MsC1KNOX1* and *MsC1KNOX2* expression was also detected in the margin of developing leaves (Figure 2c) and in a punctate pattern along the margin as the pinnae primordia arise (Figure 3d,e). We detected a scattered *MsH4* expression in the leaf apical initial, immediate derivative cells, and in some procambial cells of the developing leaf primordium, confirming that a multicellular apical region of the leaf is undergoing active cell division (Figure 3f).

Pinnae primordia emerge laterally on the developing leaf (Figure 4a). The pinna primordium has in its apex grouped cells instead of a distinct single apical cell like the one that occurs in the LAM (Figure 4b). The central region of the pinna, where the vasculature of the costa will develop, also shows an evident expression of *MsH4*, as well as the grouped apical cells (Figure 4c). The expression of *MsC1KNOX1* and *MsC1KNOX2* is detected in all the regions of the pinnae primordia at the beginning of their development (Figure 4d–f) but is gradually reduced in the abaxial region in the older developing primordia (Figure 4g,h). The expression of *MsH4* also decreases in the abaxial region of the older pinnae primordia, indicating an earlier cessation of cell division in the abaxial region compared to the adaxial region of the pinnae (Figure 4i). In the older pinnae primordia, *MsH4* expression is concentrated in marginal cells (Figure 4j). In an anatomical analysis of the pinna primordium, it is possible to detect cell divisions in the central vascular system and a denser cytoplasm in the adaxial side when compared to the more vacuolized abaxial side, also revealing a late development of the adaxial region (Figure 4k). The marginal cells of the pinna primordium are pyramidal, with an outer lenticular face, and are bigger than other cells, making them remarkably similar to leaf apical cells in transverse sections, with two cutting faces responsible for abaxial and adaxial divisions (Figure 4k). Marginal cells are organized in longitudinal rows with other cutting faces that may play some role in the proximodistal growth (Figure 4l). However, based on our experiments, it is likely that the divisions responsible for the growth in length of the pinnae take place mainly in the apical region (acropetal growth), and marginal cells act later in development, being more responsible for cell divisions that will contribute to the lamina development.

Our initial hypothesis was that *Class I KNOX* expression was stronger and longer in the developing leaves of the climbing form (that are bigger and bear more pinnae, supposedly indeterminate for a longer time), when compared with the developing leaves of the terrestrial form. Because we did not find differences in the *Class I KNOX* expression patterns in any tissues between the two forms during our ISH analysis, we measured the relative expression of *Class I KNOX* through qRT-PCR experiments (Figure S3, Supplementary Table S2). Since our study species was collected in the wild, we had limited material and also pooled individuals for each sample. Our preliminary analyses suggest that the relative expression amongst the four different samples (developing leaves of terrestrial form, shoot apices of terrestrial form, developing leaves of climbing form, and shoot apices of climbing form) is significantly different for each gene (ANOVA; *MsC1KNOX1 p* = 0.0037 and *MsC1KNOX2 p* = 0.0278; Supplementary Tables S3 and S4). A Tukey test showed that the relative expression of *MsC1KNOX1* is significantly different between the sample containing developing leaves in the terrestrial form compared to all the other analyzed samples (Supplementary Table S3). However, the Tukey test showed that the relative expression of *MsC1KNOX2* was only significantly different between the sample containing the developing leaves of the terrestrial and climbing forms (Supplementary Table S4).

## 3. Discussion

Four main conclusions can be made from our results presented here: (i) there is a multicellular structure at the tip of developing shoots, leaves, and pinnae expressing *Class I KNOX* and bearing dividing cells (based on *MsH4* expression) that may include or not (for pinnae apices) a prominent apical cell; (ii) despite the differences in the overall size and morphology of the leaves of climbing and terrestrial forms, their pattern of development is similar, possibly differing only in how long the determination is delayed; (iii) a reduction in the size and number of pinnae—interpreted by us as the result of earlier determination—is possibly correlated to a reduction in *Class I KNOX* expression; (iv) fern leaves have two types of meristems, LAM and MM, in part specified by Class I KNOX that are integral for leaf development.

The anatomical structure of shoots, leaves, and pinnae apices is very similar. In addition, all of these meristems express *Class I KNOX*. The main differences between them are the presence of single apical cells (absent in the pinnae apices) and their number of dividing planes (three in the SAM apical cell and two in the LAM apical cell). The absence of a distinct apical cell in the apices of pinnae primordia cannot be interpreted as an absence of meristems. Although more studies concerning other genes and leaf morphologies are necessary to expand this conclusion to other fern groups, our data indicates the presence of a transient apical meristem in the pinnae of *Mickelia scandens* without the presence of a prominent apical cell. The widespread reference to a unicellular meristem for ferns by some authors may be the result of many textbooks that describe in detail the single apical cell and its cutting faces, while lacking further information about the other meristematic cells in this group (e.g., [22,38,39,40]), contributing to the propagation of this concept. A well-documented work that strongly defended the idea of a single-celled meristem was based on the observation of apical cell division planes in more than 50 genera of ferns [21]. However, several authors [17,18,41,42] proposed cytohistological zonation schemes for a multicellular structure, based mainly on the fact that the apical initial cell rarely divides. Based on the *Class I KNOX* expression data in *Elaphoglossum peltatum* f. *peltatum* and reviewing these previous studies, a recent study proposed a simplified zonation for the shoot apical meristem of ferns: a single apical cell that rarely divides and may not express *Class I KNOX* genes in some apices, and a peripheral zone with rapidly dividing cells [20]. It is important to highlight that even with data supporting a reduced mitotic activity for the single apical cells, they can still divide in the SAM and LAM and are likely the ultimate source of all cells similar to the quiescent center of seed plant meristems. The significance of the occasional absence of *Class I KNOX* expression in the apical cells is not clear; however, these shoots were still active, as *Class I KNOX* genes were detected in the peripheral zone of the SAM.

Our preliminary qRT-PCR experiments are the first ones to show that, in ferns, lower *Class I KNOX* expression is possibly correlated to a more determinate structure. These data support the conclusion that, based on *Class I KNOX* expression patterns, complex leaves should be interpreted as partially indeterminate structures [7]. However, more adequate conditions are needed to confirm this possibility, such as biological replicates and the use of plants grown in very controlled conditions, as well as exploring the expression of Class *I KNOX* genes in other ferns with different leaf forms. Further studies exploring the phenotype of fern mutants for Class *I KNOX* are also needed to test our hypothesis when they become available. In the *Mickelia scandens* developing leaf, *Class I KNOX* genes are expressed throughout the apical region, encompassing the apical cell to the cells of the first pinnae primordia and reinforcing the presence of a multicellular apical meristem in the leaf, similar to the shoot apex. The expression of *Class I KNOX* in the pinnae primordium, even as a terminal unit, suggests some degree of indeterminacy. This is reinforced by the anomalous leaf (Figure 1) that resembles other species of the genus, *Mickelia furcata* R.C. Moran, Labiak and Sundue, a plant with bipinnate leaves at the basal pinnae [33]. Possibly, a plant overexpressing *Class I KNOX* will show a similar phenotype. Additionally, cell division patterns at the pinna apex together with *Class I KNOX* expression suggest a meristematic activity in this region, even though those pinnae apices do not have an evident single apical cell or smaller derivative cells (Figure 4c,d). After the initial acropetal growth, the abaxial differentiation occurs preceding the adaxial differentiation, a phenomenon well-known for flowering plants [43]. In ferns, this may be responsible for the typical coiling of young fern leaves (known as a fiddlehead or crozier), thus protecting these meristematic apices of the leaf and pinnae.

Fern apical meristems should be interpreted as a complex and highly organized interconnected network of cells with indeterminate fates, specialized zones (apical cells vs. peripheral cells), and the capacity for producing new organs (leaves or pinnae). Interestingly, many studies have interpreted fern leaves as reiterative and fractal systems, in which the shoot apices generate structures that can repeat some degree of their own shoot development [19,44,45]. In this sense, as already have been stated by some authors [26,46,47], fern leaves and their segments could be interpreted evolutionarily and ontogenetically as reduced shoots, and the presence of similar characteristics detected by us in *Mickelia scandens* (i.e., *Class I KNOX* expression during initial development and cell divisions concentrated in apical and surrounding cells) gives support for this interpretation. The presence of such features in developing leaves is strong evidence that Agnes Arber’s Partial Shoot Theory [28,29] is correct. Arber said that “the leaf is a partial-shoot, arising laterally from a parent whole-shoot”, based mainly on the presence of lateral structures arising from axial elements in the leaf, as well as in shoots. According to her, the shoot has a gradient of determination between stems and leaves, and compound leaves present the same gradient. Her theory should be strongly discussed now that new molecular evidence, as our results and other studies discussed here, is available. Our results point to multicellular meristematic structures in the shoot, leaf, and pinna apices, also reinforcing her idea of “identity-in-parallel”, in which structures may be put in a relation of the part to the whole, but is also equivalent as a whole [28]. The pinna is part of the shoot, but ultimately is equivalent to a whole shoot, carrying the potential of producing new lateral structures. The observed anomalous pinna with a lateral segment (Figure 1k) could be evidence of this potential.

The future of fern studies is promising, as new sequences are available in transcriptome projects like oneKP [48] and the first fern genomes are already available for *Salvinia cucullata* Roxb. and *Azolla filiculoides* Lam. [49,50]. The discovery of fern genes related to apical meristems and their regulation will certainly increase our understanding and can even detail better the zonation and functions of different cell niches. New developmental studies with multiple approaches, uniting these modern molecular analyses with classical anatomical data for developmental studies in ferns will certainly help us to better understand the evolution of all leaves.

## 4. Materials and Methods

### 4.1. Plant Material

Shoot apices (usually containing small leaf primordia covered by scales) and developing leaves of the terrestrial and climbing forms of *Mickelia scandens* sporophytes were collected from specimens that occur in a dense population in Fontes do Ipiranga State Park (São Paulo, Brazil). A voucher specimen is deposited in the SP Herbarium (Prado and Cruz 2332). Part of the material was stored in RNAlater^®^ for RNA extraction and some were fixed in formalin-acetic acid-ethanol 50% (FAA) for in situ hybridization (ISH) experiments and anatomy.

### 4.2. RNA extraction and cDNA Synthesis

The total RNA of the shoot apices and developing leaves was extracted with QIAGEN RNeasy mini kit (Qiagen, Hilden, Germany). A cDNA synthesis was performed using Superscript III (Invitrogen, Carlsbad, CA, USA), following the manufacturers’ protocols for these procedures (except for qRT-PCR). The cDNA for qRT-PCR was obtained with SuperScript IV VILO Master Mix (Invitrogen, Carlsbad, CA, USA).

### 4.3. Genes Isolation and Phylogenetic Analyses

Degenerate primers were used for KNOX genes (F: 5′ -CCBGARCTBGACMABTTYATGG-3′, R: 5′-CCAGTGSCKYTTCCKYTGRTTDATRAACC-3′), based on a previous study [20] for H4 genes (F: 5′-ATGTCWGGMMGRGGWAAGGGAGG-3′, R: 5′-CCRAADCCRTARAGVGTHCKKCC-3′) designed for this study to be used as a cell division marker, as used in previous studies [51,52]. Fragments were cloned in Invitrogen^TM^ pCR^TM^ 2.1–TOPO^TM^ 3.9 kb plasmids and sequenced with M13 primers. The sequences (Supplementary Table S1) were then analyzed by the NCBI Conserved Domain Search tool [53] to detect the presence of KNOX and H4 domains. Unlike Class I, the phylogenetically distinct Class II KNOX that is also a target of these primers is related to tissue differentiation and not to cell proliferation in land plants [3]. In order to identify our cloned *KNOX* fragments, the sequences were aligned with other known *KNOX* genes (sequences referenced in two previous studies) [3,20] with Geneious version 10.1.2 [54]. Phylogenetic relationships were inferred from the nucleotide data using maximum likelihood (ML) analyses. ML searches for the best tree and bootstrap were performed simultaneously with 300 replicates with RaxML version 8 [55], partitioned by codon position with GTR + Γ + I model as recommended in a PartitionFinder2 analysis [56].

### 4.4. Anatomy and In Situ Hybridization (ISH) Experiments

Fixed material was embedded in paraplast (Fisher) and sectioned on a rotary microtome. For histological analyses, sections were stained with Safranin O 1% in ethanol, Crystal Violet 1% aqueous and Orange G 1% in clove oil [57]. For the ISH experiments, we followed the procedures previously described [58,59] using specific probes for *Class I KNOX* and *H4* generated with specific primers designed for them (Supplementary Table S5). The similarity between probes is 57% in *Class I KNOX* genes (Supplementary Figure S1).

### 4.5. Quantitative Real-Time PCR

In order to quantify the expression of *Class I KNOX* genes in the shoot apices and developing leaves of terrestrial and climbing forms, we assessed the transcript abundance by a qRT-PCR analysis using a 7500 Real-Time PCR system (Applied Biosystems^®^ by Life Technologies, NY, USA). A β-actin specific sequence was accessed with PCR reactions with primers (F: 5′-GATGGATCCTCCAATCCAGACACTGTA-3′ and R: 5′-GTATTGTGTTGGACTCTGGTGATGGTGT-3′) and was used as a housekeeping gene. The PCR reactions were performed with 5 µl of cDNA; 12.5 µl of SYBR Green Master Mix (Applied Biosystems); 10 pmol/µl concentration of primers (specifically designed for qRT-PCR analysis, Supplementary Table S5); and the following cycling conditions: 95 °C for 10 min, 44 cycles of 95 °C for 15 s, 55 °C for 30 s and 72 °C for 1 min. All the reactions were performed in three technical replicates, each one analyzing the expression in four different samples: the developing leaves of the terrestrial form, the shoot apices of the terrestrial form, the developing leaves of the climbing form, and the shoot apices of the climbing form. Each sample was extracted from a single pool of material containing at least five different individuals randomly collected from the population. The expression was calculated using the ∆*C*_T_ (difference between threshold cycles) method [60], and the statistical significance was determined with ∆*C*_T_ values by using a one-way ANOVA test followed by Tukey’s pairwise comparison (*p* < 0.05). This preliminary analysis can detect only that these four samples have different relative gene expression, potentially underestimating some of the differences due to biological variations between different individuals within the population. Raw data and calculations are available (Supplementary Tables S2–S4).

## Supplementary Materials

Supplementary Materials can be found at https://www.mdpi.com/1422-0067/21/12/4295/s1. Figure S1, Alignment of partial KNOX sequences with conserved domains and probe binding sites; Figure S2, Phylogenetic tree showing relationships between KNOX genes; Figure S3, Relative expression of Class I KNOX genes in different pools; Table S1, GenBank accession numbers of obtained sequences; Table S2, Ct values of qRT-PCR and relative expression calculation; Table S3, ANOVA and Tukey’s pairwise comparison of MsC1KNOX1 expression in different tissues; Table S4, ANOVA and Tukey’s pairwise comparison of MsC1KNOX2 expression in different tissues; Table S5, Primer sequences.

## Abbreviations

*Class I KNOX*	*Class I KNOTTED-LIKE HOMEOBOX*
ISH	In-situ hybridization
LAM	Leaf apical meristem
MM	Marginal meristem
qRT-PCR	Real-time quantitative reverse transcription polymerase chain reaction
SAM	Shoot apical meristem
