# Supplementary material for: Class I KNOX Is Related to Determinacy during the Leaf Development of the Fern Mickelia scandens (Dryopteridaceae)
Source: Int J Mol Sci. 2020 Jun 16;21(12):4295. doi: 10.3390/ijms21124295 (PMC7352642; doi:10.3390/ijms21124295)
Supplement: Supplementary file 1 [file ijms-21-04295-s001.zip › Supplementary/Supplementary Figures.pdf]

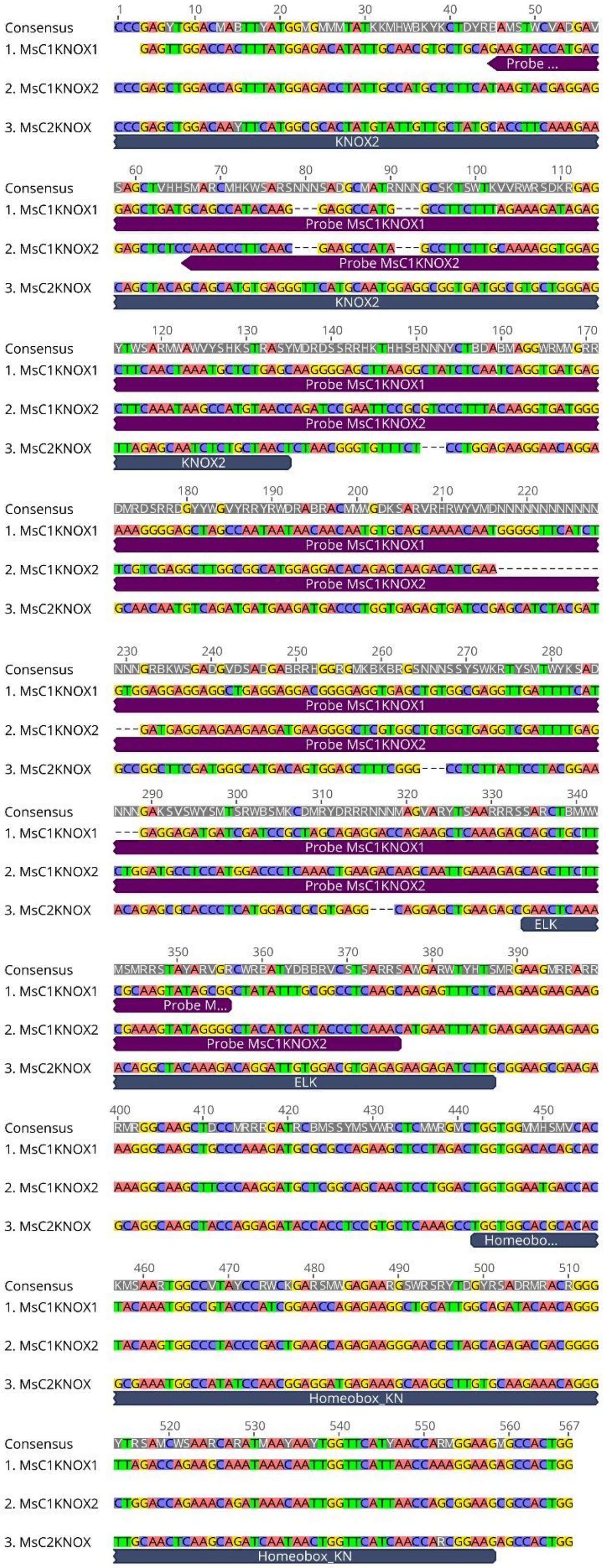

Figure S1. Alignment of partial *KNOX* sequences with conserved domains and probe binding sites.

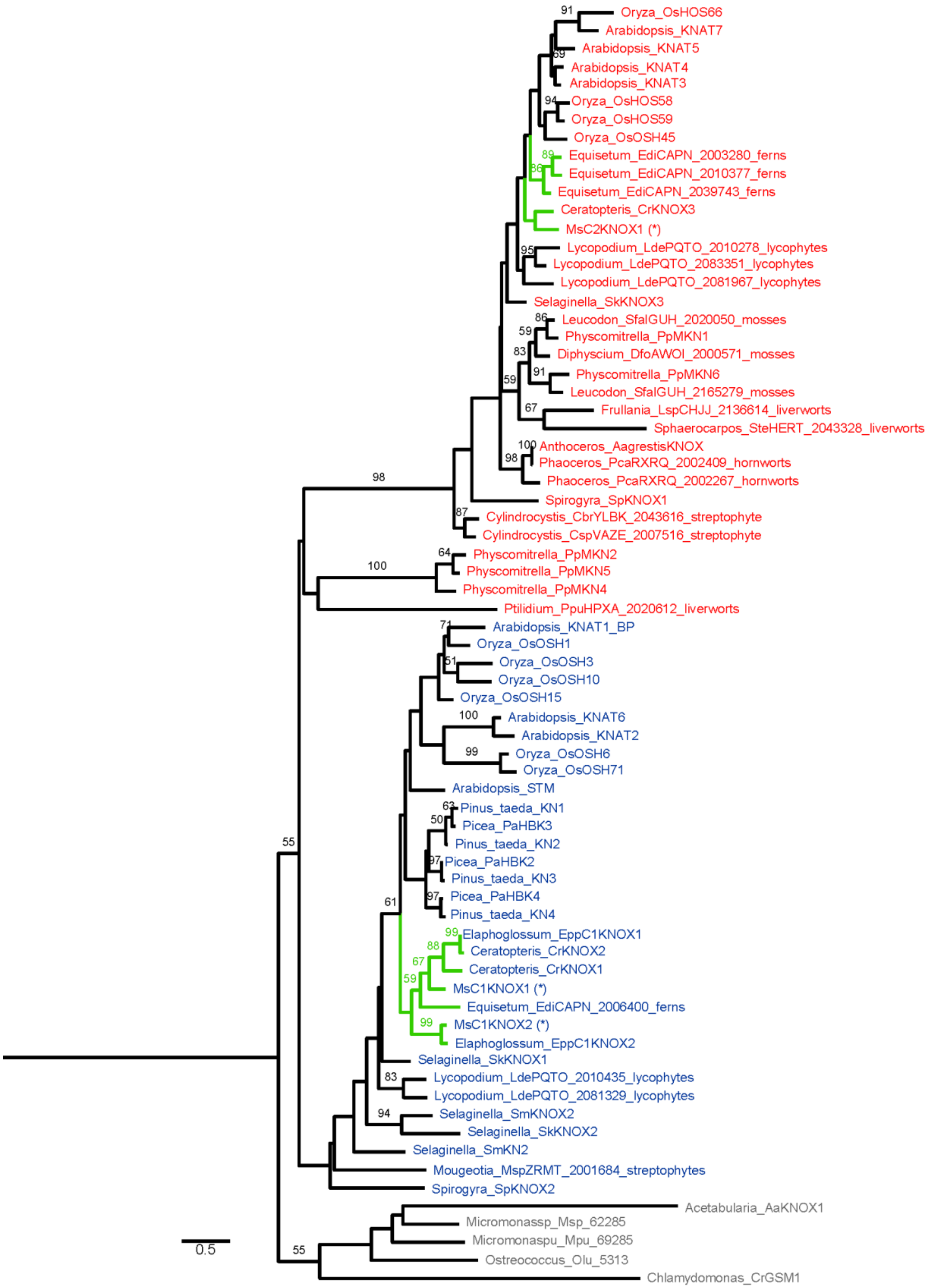

Figure S2. Phylogenetic tree showing relationships between *KNOX* genes.

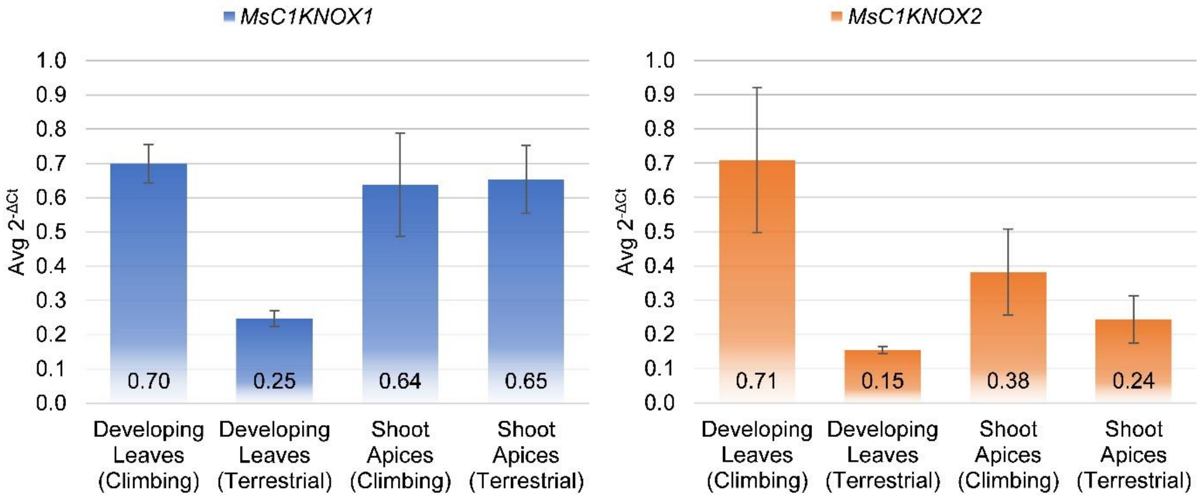

Figure S3. Relative expression levels ± SE of *Class I KNOX* genes in analyzed pools of tissues of *Mickelia scandens*. Expression level of *MsC1KNOX1* is significantly different between the single analyzed pool containing developing leaves in the terrestrial form compared to all other analyzed pools (Tukey's pairwise $p<0.05$, Supplementary Table S3). *MsC1KNOX2* expression is only significantly different between the pool containing developing leaves of the terrestrial and the pool containing climbing forms (Tukey's pairwise $p<0.05$, Supplementary Table S4).
