# Supplementary material for: Class I KNOX Is Related to Determinacy during the Leaf Development of the Fern Mickelia scandens (Dryopteridaceae)
Source: Int J Mol Sci. 2020 Jun 16;21(12):4295. doi: 10.3390/ijms21124295 (PMC7352642; doi:10.3390/ijms21124295)
Supplement: Supplementary file 1 [file ijms-21-04295-s001.zip › Supplementary/Supplementary Table S1_May24_FINAL.doc]

**Table S1.** GenBank accession numbers of obtained sequences.

| **Sequence** | **GenBank Accession** |
| --- | --- |
| *MsC1KNOX1* | MH668283 |
| *MsC1KNOX2* | MH668284 |
| *MsC2KNOX* | MH668285 |
| *MscandensBActin* | MH668286 |
| *MscandensH4* | MH668287 |
