# Supplementary material for: Class I KNOX Is Related to Determinacy during the Leaf Development of the Fern Mickelia scandens (Dryopteridaceae)
Source: Int J Mol Sci. 2020 Jun 16;21(12):4295. doi: 10.3390/ijms21124295 (PMC7352642; doi:10.3390/ijms21124295)
Supplement: Supplementary file 1 [file ijms-21-04295-s001.zip › Supplementary/Supplementary Table S5_May24_FINAL.doc]

**Table S5. Primer sequences (5’🡪3’).**

| **Primers designed for probes labeling:** | |
| --- | --- |
| MsC1KNOX1ProbeF | GAAGTACCATGACGAGCTGATGC |
| MsC1KNOX1ProbeR | CCGCTATACTTGCGAAGCAG |
| t7_MsC1KNOX1ProbeR | CTAATACGACTCACTATAGGGCCGCTATACTTGCGAAGCAG |
| MsC1KNOX2ProbeF | CAAACCCTTCAACGAAGCCATA |
| MsC1KNOX2ProbeR | GTTTGAGGGTAGTGATGTAGC |
| t7_MsC1KNOX2ProbeR | CTAATACGACTCACTATAGGGGTTTGAGGGTAGTGATGTAGC |
| MscandensH4ProbeF | ATGTCTGGCCGGGGAAAGGGAGG |
| MscandensH4ProbeR | CCGAAGCCATAGAGGGTACTTCC |
| t7_MscandensH4ProbeR | CTAATACGACTCACTATAGGGCCGAAGCCATAGAGGGTACTTCC |
| **Primers designed for qRT-PCR experiments:** | |
| MsC1KNOX1qRTF | GTGATGAGAAAGGGGAGCTAG |
| MsC1KNOX1qRTR | GCCTCCTCCTCCACAGATGA |
| MsC1KNOX2qRTF | GACACAGAGCAAGACATCGA |
| MsC1KNOX2qRTR | TTCAGTTTGAGGGTCCATG |
| MsActinqRTF | CGTCTGGATCTTGCTGGC |
| MsActinqRTR | CTCGCTCAGCAGTGGTT |
